# Antitumor effectiveness of different amounts of electrical charge in Ehrlich and fibrosarcoma Sa-37 tumors

**DOI:** 10.1186/1471-2407-4-87

**Published:** 2004-11-26

**Authors:** HC Ciria, MS Quevedo, LB Cabrales, RP Bruzón, MF Salas, OG Pena, TR González, DS López, JM Flores

**Affiliations:** 1 Sección de Bioelectricidad. Departamento de Bioingeniería y Equipos, Centro Nacional de Electromagnetismo Aplicado, Universidad de Oriente, Santiago de Cuba 90400, Cuba; 2 Hospital Oncológico Docente Provincial Conrado Benítez, Santiago de Cuba 90100, Cuba; 3 Departamento de Inmunología, Hospital Provincial Clínico Quirúrgico Docente Saturnino Lora, Santiago de Cuba 90500, Cuba; 4 Hospital Infantil Norte Docente "Juan Martínez de la Cruz Maceira". Santiago de Cuba, Cuba; 5 Dirección Municipal de Salud Pública. Santiago de Cuba, Cuba; 6 Departamento de Investigación en Física, Universidad de Sonora, Apdo. Postal A-088, 83190 Hermosillo, Sonora, México

## Abstract

**Background:**

*In vivo *studies were conducted to quantify the effectiveness of low-level direct electric current for different amounts of electrical charge and the survival rate in fibrosarcoma Sa-37 and Ehrlich tumors, also the effect of direct electric in Ehrlich tumor was evaluate through the measurements of tumor volume and the peritumoral and tumoral findings.

**Methods:**

BALB/c male mice, 7–8 week old and 20–22 g weight were used. Ehrlich and fibrosarcoma Sa-37 cell lines, growing in BALB/c mice. Solid and subcutaneous Ehrlich and fibrosarcoma Sa-37 tumors, located dorsolaterally in animals, were initiated by the inoculation of 5 × 10^6 ^and 1 × 10^5 ^viable tumor cells, respectively. For each type of tumor four groups (one control group and three treated groups) consisting of 10 mice randomly divided were formed. When the tumors reached approximately 0.5 cm^3^, four platinum electrodes were inserted into their bases. The electric charge delivered to the tumors was varied in the range of 5.5 to 110 C/cm^3 ^for a constant time of 45 minutes. An additional experiment was performed in BALB/c male mice bearing Ehrlich tumor to examine from a histolological point of view the effects of direct electric current. A control group and a treated group with 77 C/cm^3 ^(27.0 C in 0.35 cm^3^) and 10 mA for 45 min were formed. In this experiment when the tumor volumes reached 0.35 cm^3^, two anodes and two cathodes were inserted into the base perpendicular to the tumor long axis.

**Results:**

Significant tumor growth delay and survival rate were achieved after electrotherapy and both were dependent on direct electric current intensity, being more marked in fibrosarcoma Sa-37 tumor. Complete regressions for fibrosarcoma Sa-37 and Ehrlich tumors were observed for electrical charges of 80 and 92 C/cm^3^, respectively.

Histopathological and peritumoral findings in Ehrlich tumor revealed in the treated group marked tumor necrosis, vascular congestion, peritumoral neutrophil infiltration, an acute inflammatory response, and a moderate peritumoral monocyte infiltration. The morphologic pattern of necrotic cell mass after direct electric current treatment is the coagulative necrosis. These findings were not observed in any of the untreated tumors.

**Conclusion:**

The data presented indicate that electrotherapy with low-level DEC is feasible and effective in the treatment of the Ehrlich and fibrosarcoma Sa-37 tumors. Our results demonstrate that the sensitivity of these tumors to direct electric current and survival rates of the mice depended on both the amount of electrical charge and the type of tumor. Also the complete regression of each type of tumor is obtained for a threshold amount of electrical charge.

## Background

The use of electric current in the treatment of malignant tumors has been known since the beginning of the 19^th ^century. Several investigators have reported encouraging results from experimental low-level direct current therapy (DEC) in different types of tumor [1-3]. These studies have shown that DEC has an antitumor effect in different animal tumor models and in clinic; however, it has not yet been universally accepted.

The dose-response relationships obtained in these studies indicate that the DEC effectiveness depends on both the type of tumor and therapeutic scheme (amount of electrical charge and electrode array). Lack of guidance has become an obstacle to introduce the electrochemical treatment (EChT) in the clinic oncology. This is due to the lack of standardization of the EChT method regarding DEC doses and electrode array. Ren et al. [4] studied the influence of the dose and electrode spacing in the breast cancer and concluded that an increase of the dose lead to an increase in both the necrosis percentage and increased survival rate. However, they did not find significant spacing effect on the tumor necrosis percentage. On the other hand, Chou et al. [5] revealed that the number of electrodes depends on the tumor size and that the electrodes inserted at the base perpendicular to the tumor long axis increased the antitumor effectiveness respect to other electrode configurations used.

In spite of these results, the efficacy of DEC treatment has been controversial since an optimum electrode array and a threshold amount of electrical charge for each type of tumor have not been established. We believe that the procedure to determine the amount of electrical charge for each type of tumor is completely destroyed is more feasible to implement than that for the optimum electrode array, which involves several variables, such as polarity, number, and orientation of the electrodes. The knowledge of the optimum values of these parameters may lead to maximize the antitumor effectiveness of DEC and minimize their adverse effects in the organism. This allows the establishment of a therapeutic procedure for the tumor treatment in animals and in clinical oncology.

The aim of this study is to test the hypothesis that the responses of the tumors treated with DEC is dependent on dose. Ehrlich and fibrosarcoma Sa-37 tumors were used. The survival rates of the mice bearing of these two types of tumor were determined. The antitumor effects of DEC were also evaluated through the peritumoral and tumoral findings in Ehrlich tumor.

## Methods

### Animals

The experiment was run in accordance with Good Laboratory Practice rules and animals protection laws. The experiment was approved by the ethical committee of Oriente University, which follows the guideline from the Cuban Animal Ethical Committee. BALB/c male mice, 7–8 week old and 20–22 g weight were used. They were supplied from the National Center for Production of Laboratory Animals (CENPALAB), Havana City, Cuba, and were kept in standard laboratory conditions with water and food ad libitum. Animals were healthy (without signs of fungal or other infections) and were maintained in plastic cages inside a room at a constant temperature of 23 ± 2°C and relative humidity of 65 %, and a natural day-night cycle. During therapy the animals were firmly fixed on wooden boards, so all treatments were performed in the absence of anesthesia. All treated animals showed uneasy and quick breathing during fixation.

### Tumor cell lines

Ehrlich and fibrosarcoma Sa-37 cell lines, growing in BALB/c mice, were received from the Center for Molecular Immunology, Havana City, Cuba. Both cell lines are being maintained in the Cell Culture Collection of the Department of Pathologic Anatomy, Hospital "Conrado Benítez", Santiago de Cuba, Cuba.

The Ehrlich and fibrosarcoma Sa-37 ascitic tumor cell suspensions, transplanted to the BALB/c mouse, were prepared from the ascitic forms of the tumors. Ehrlich solid and subcutaneous tumors, located dorsolaterally in animals, were initiated by the inoculation of 5 × 10^6 ^viable tumor cells in 0.2 ml of 0.9 % NaCl, while fibrosarcoma Sa-37 solid and subcutaneous tumors located dorsolaterally in animals, were initiated by the inoculation of 1 × 10^5 ^viable tumor cells in 0.2 ml of 0.9 % NaCl. For both tumors, the viability of the cells was determined by Trypan blue dye exclusion test and it was over 95 %. Cell count was made using an hematocytometer.

Tumor growth was followed by measuring three perpendicular tumor diameters (a, b and c, where a > b > c) with a vernier caliper. The tumor volume was estimated using the equation 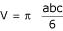. The mean tumor volume with the corresponding standard deviation of three determinations was calculated in each experimental group. Mice with non-palpable tumor at day 60 after the treatment were designated as cured.

Tumor doubling time (DT, in *days*) was determined for each individual tumor as the time needed to double the initial tumor volume. For each experimental group the mean DT and its standard deviation were calculated.

### Histopathological study of the Ehrlich tumor

The histologic cuts from each tumor were made according to the largest diameter. They were fixed in a 10 % formol solution and processed by the paraffin method.

Hematoxylin and eosin stained slides were used to evaluate the presence of necrosis. Hematoxylin and eosin stained slides were examined under an Olympus light microscope. The extent of necrosis was defined as the percentage of necrotic region compared with the whole area of the tumor section.

The peritumoral alterations were evaluated as none (-), slight (+), moderate (++) and severe (+++).

### Electrochemical treatment

To supply electrochemical treatment, a high stability and low noise DEC source was built at the National Center for Applied Electromagnetism (CNEA). The electrode configuration consisted of a multi-electrode array formed by two anodes and two cathodes inserted into the base perpendicular to the tumor long axis keeping about 3 mm distance between them. Cathode and anode were connected in alternate sequence. This multi-electrodes array was proposed taking into account the results reported by Chou et al. [5]. All electrodes were cleaned and sterilized in alcohol prior to use. Platinum electrodes of 0.7 mm diameter and 20 mm long were used. After the electrodes were inserted, they were connected to the DEC source.

In order to find the thresholds of the electrical charge for which Ehrlich and fibrosarcoma Sa-37 tumors are completely destroyed, different amounts of electrical charge in the range of 5.5 to 110 C/cm^3 ^were used. From this range of electrical charge three values were chosen to show the DEC effectiveness in both types of tumors. When the tumors reached approximately 0.5 cm^3 ^in BALB/c mice, a single shot electrotherapy was supplied (zero day). For each type of tumor four groups consisting of 10 mice each randomly divided were formed. For Ehrlich tumor the groups formed were: control group (CG1), treated group with electrical charge of 36 C/cm^3 ^(18.0 C in 0.5 cm^3^) and 6.7 mA for 45 min (TG1-1), treated group with 63 C/cm^3 ^(31.5 C in 0.5 cm^3^) and 11.7 mA for 45 min (TG1-2), and treated group with 92 C/cm^3 ^(46.0 C in 0.5 cm^3^) and 17 mA for 45 min (TG1-3). For fibrosarcoma Sa-37 tumor the groups formed were: control group (CG2), treated group with 36 C/cm^3 ^(18 C in 0.5 cm^3^) and 6.7 mA for 45 min (TG2-1), treated group with 63 C/cm^3 ^(31.5 C in 0.5 cm^3^) and 11.7 mA for 45 min (TG2-2), and treated group with 80 C/cm^3 ^(40.0 C in 0.5 cm^3^) and 14.8 mA for 45 min (TG2-3).

The dose of 105 C/cm^3 ^(52.5 C in 0.5 cm^3^) and 19.4 mA for 45 min was supplied to 10 mice (5 mice bearing Ehrlich tumor and 5 mice bearing fibrosarcoma Sa-37 tumor). Also the dose of 110 C/cm^3 ^(55 C in 0.5 cm^3^) and 20.3 mA for 45 min was supplied to 10 mice (5 mice bearing Ehrlich tumor and 5 mice bearing fibrosarcoma Sa-37 tumor). These doses were used to evaluate the therapeutic and adverse effects of the DEC above 100 C/cm^3^. For each type of tumor was formed a control group of 10 mice.

In order to examine from the histolological point of view the effects of direct electric current in Ehrlich tumor two experimental groups were formed: a control group (CG-A) and a treated group with 77 C/cm^3 ^(27.0 C in 0.35 cm^3^) and 10 mA for 45 min (TG-A). This treated group was divided in three subgroups TG1-A, TG2-A and TG3-A to show the tumor and peritumoral findings at 1, 2 and 4 days after DEC treatment. Each experimental group was formed by 6 mice. When the Ehrlich tumor volumes reached 0.35 cm^3^, two anodes and two cathodes were inserted into the base perpendicular to the tumor long axis and a single shot electrotherapy was supplied (zero day).

In all experiments, before treatment the DEC was increased gradually step by step for two minutes until the desired intensity. During treatment it was constant and continually monitored. The voltage was also continually monitored. It varied, in accordance with the change of tissue resistance during the current application, between 5 and 25 V. The total electrical charge was calculated in real time. After a single application of the intended dose, the treatment was stopped. In this case, the current was decreased step by step for two minutes until its intensity was 0 mA. During electrotherapy, mice were firmly restrained, without obvious discomfort; therefore no anesthesia was necessary.

In the control groups, four electrodes were placed into the base perpendicular to the tumor long axis without applying any direct current (0 mA). The animals of this group were firmly fixed but without DEC and showed uneasy and quick breathing during their fixation.

Survival rates of the mice bearing both types of the tumors were determined for each experimental group. The survival rate (in %) was defined as the ratio between the number of live animals and the total number of animals, multiplied by 100 %. Survival checks mortality were made daily.

### Histopathological study of the tumor

The histologic cuts from each tumor were made according to the largest diameter. They were fixed in a 10 % formal solution and processed by the paraffin method.

Hematoxylin and eosin staining was used. Each cut was divided into four microscopic fields in order to calculate the necrosis percentage through panoramic lens. This percentage was calculated as the ratio between the necrosis area and the tumor total area, multiplied by 100 %.

### Statistical criteria

The nonparametric statistical criterion of one-tailed Wilcoxon-Mann-Whitney rank sum was used to compare volumes between the treated groups with DEC and their respective control groups. Survival curves for the three different mice treatment groups for each tumor type were estimated by using the Kaplan-Meier product limit estimator [6].

McNemar's statistical criterion was used for comparing the main histopathological findings in peritumoral zones in animals from CG-A and TG-A. P values of less than 0.05 were considered significant. The mean value and its mean standard error were reported for each experimental group.

## Results

**Figure 1:**
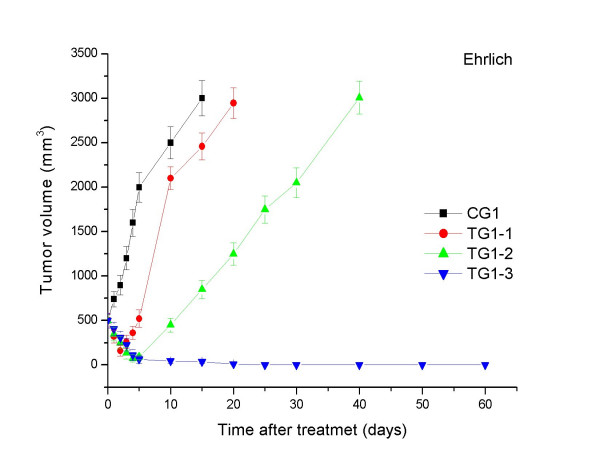
**Effect of DEC on the growth curve of Ehrlich tumor**. Data are means ± mean standard error (vertical bars). The experimental groups formed for the Ehrlich tumor were control group, CG1 (-■-); treated group with 36 C/cm^3^, TG1-1 (-●-); treated group with 63 C/cm^3^, TG1-2 (-▲-); treated group with 92 C/cm^3^, TG1-3 (-▼-). Each experimental group is formed by 10 mice.

**Figure 2:**
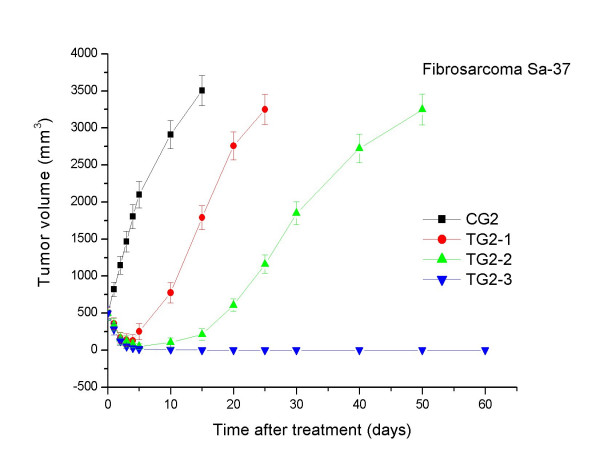
**Effect of DEC on the growth curve of fibrosarcoma Sa-37 tumor**. Data are means ± mean standard error (vertical bars). The experimental groups formed for the fibrosarcoma Sa-37 tumor were control group, CG2 (-■-); treated group with 36 C/cm^3^, TG2-1 (-●-); treated group with 63 C/cm^3^, TG2-2 (-▲-); treated group with 80 C/cm^3^, TG2-3 (-▼-). Each experimental group is formed by 10 mice.

As it is shown in Table 1 and Figure 1, Ehrlich tumors in DEC-treated mice were significantly inhibited as compared with tumors of untreated mice (P < 0.02). This tumor growth inhibition following DEC treatment was observed in every individual mouse. Also there are significant differences between the treated groups being more evident for TG1-3 (P < 0.05). Similar effect of DEC treatment was observed in fibrosarcoma Sa-37 bearing mice (Table 1 and Fig. 2). In these mice DEC treatment also resulted in significant inhibition of tumor growth (P < 0.02). For this type of tumor also were observed significant differences between the treated groups being more evident for TG2-3 (P < 0.05).

The results shown in this study revealed that the sensitivity of the Ehrlich and fibrosarcoma Sa-37 tumors was dose dependent. The sensitivity to DEC of both types of tumors increased with the increase of the amount of electrical charge (Table 1 and Figs. 1 and 2). These results also made evident that fibrosarcoma Sa-37 tumor were more sensitive to DEC than Ehrlich tumor under the same amount of electrical charge (TG1-1 compared with TG2-1 and TG1-2 compared with TG2-2). For these doses there were significant differences (P < 0.05). It was also observed on Ehrlich tumor for doses of 36 and 63 C/cm^3 ^that the tumors partially regressed for 2 and 4 days, respectively. For these same doses the fibrosarcoma Sa-37 tumor reached their respective partial regressions for 4 and 5 days. Both eventually outgrew again.

The complete regression of the Ehrlich tumor was observed 25 days after treatment with 92 C/cm^3 ^(Table 1 and Fig. 1); however, for the fibrosarcoma Sa-37 tumor it was observed 15 days post-treatment with 80 C/cm^3 ^(Table 1 and Fig. 2). After 60 days post-treatment the tumors were non palpable in TG1-3 and TG2-3. For these doses there were no significant differences (P > 0.05) in the growth of these two types of tumor after treatment; however, there were significant differences in the time for which each type of tumor was completely destroyed (P < 0.05).

In the case of the untreated tumors, fibrosarcoma Sa-37 tumor showed a quicker growth than that of the Ehrlich tumor. Also, the DT of fibrosarcoma Sa-37 was 0.7 times smaller than that of the Ehrlich tumor (Table 1).

**Figure 3:**
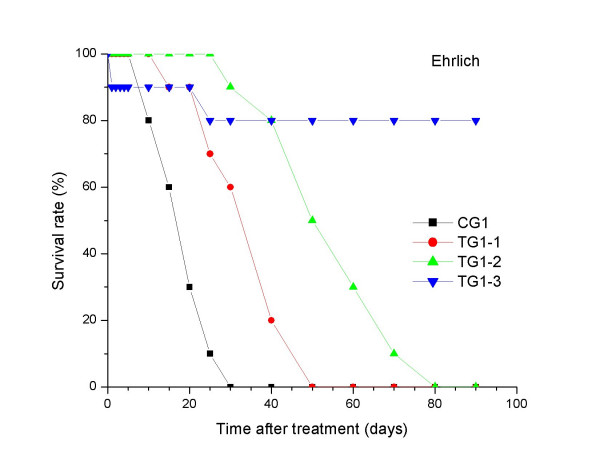
**Survival rates in BALB/c mice bearing Ehrlich tumor after electrochemical treatment**. Data are means ± mean standard error (vertical bars). The experimental groups formed for the Ehrlich tumor were control group, CG1 (-■-); treated group with 36 C/cm^3^, TG1-1 (-●-); treated group with 63 C/cm^3^, TG1-2 (-▲-); treated group with 92 C/cm^3^, TG1-3 (-▼-). Each experimental group is formed by 10 mice.

**Figure 4:**
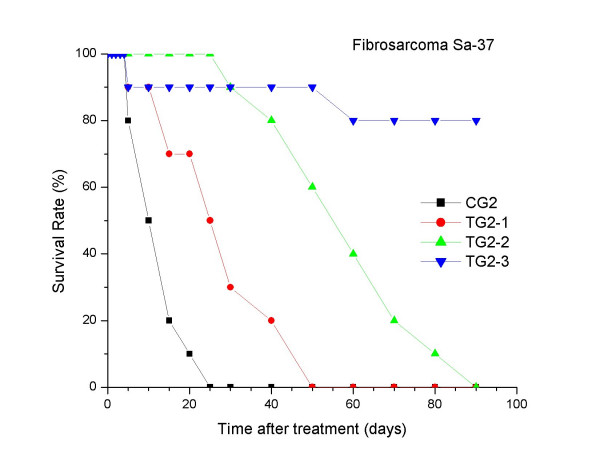
**Survival rates in BALB/c mice bearing fibrosarcoma Sa-37 tumor after electrochemical treatment**. Data are means ± mean standard error (vertical bars). The experimental groups formed for the fibrosarcoma Sa-37 tumor were control group, CG2 (-■-); treated group with 36 C/cm^3^, TG2-1 (-●-); treated group with 63 C/cm^3^, TG2-2 (-▲-); treated group with 80 C/cm^3^, TG2-3 (-▼-). Each experimental group is formed by 10 mice.

The overall survival curves of the mice bearing Ehrlich and fibrosarcoma Sa-37 tumors are shown in figures 3 and 4, respectively. These figures show that for both types of tumors the survival rate of the mice treated with DEC was significantly greater when compared with that of their respective untreated mice (P < 0.001). In this figure it was also observed that the cure rates were 80 % (8/10) for Ehrlich tumor (TG1-3) and 90 % (9/10) for fibrosarcoma Sa-37 tumor (TG2-3). Significant differences between the survival rates of the mice treated with different amounts of electrical charge (P < 0.05) were also found, being more marked for TG1-3 and TG2-3 for Ehrlich and fibrosarcoma Sa-37 tumors, respectively. For the dose of 36 C/cm^3 ^there were no significant differences between both types of tumor (P > 0.05); however, for the other doses there were significant differences (P < 0.05).

The cured mice were sacrificed at 100 days post-treatment. Before sacrifice, the animals were active and in good physical condition with adequate body weight. They had good posture and coats of hair. After sacrifice, the histopathological findings in each of these mice showed complete disappearance of the tumor and evidence of healing. In the treated mice a very little necrotic tissue remained within a fibrous scar. Serology and histological finding of the organs did reveal neither abnormalities nor metastases (results not shown).

The death of a mouse 1-day after DEC treatment was observed in TG1-3. The histological findings revealed damages in the lungs due to hemorrhage and a small circular necrosis. Metastases were not observed in this mouse. It was also observed the death of a mouse 25 days post-treatment in TG1-3 and 50 days in TG2-3 due to the cannibalism shown by the mice, probably because of the blood present in the tumors after DEC treatment. All the mice died for amounts of electrical charge above 100 C/cm^3^, during the first 24 hours after DEC treatment. The histological findings showed both severe alterations in liver and kidney and an increase in the weight of these organs. Metastases were not observed in any of these mice.

**Figure 5:**
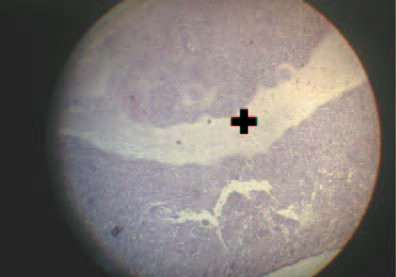
Central necrosis area in an Ehrlich untreated tumor (+). HE. × 32.

**Figure 6:**
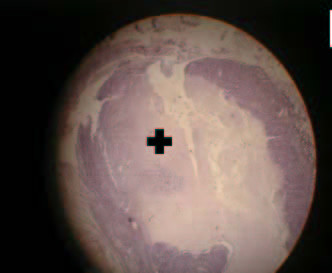
Necrosis area in the Ehrlich treated tumor (+) 2 days after DEC treatment. HE. × 32.

**Figure 7:**
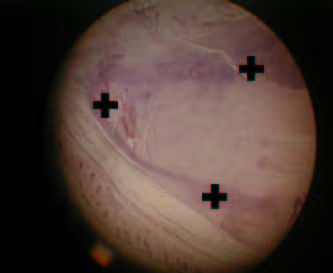
Necrosis area in the Ehrlich treated tumor (+) 4 days after DEC treatment. HE. × 32.

The histopathological findings revealed that in the Ehrlich untreated tumors (CG-A) the necrotic area was mainly central and it constituted approximately from 20 % of the tumor total area (Fig. 5). However, in tumors treated with DEC, a wide necrotic area was observed. The tumor necrosis percentages of treated groups at 1, 2 and 4 days after treatment were approximately 2.7, 3.9 (Fig. 6) and 4.7 (Fig. 7) times higher than that of the CG-A, respectively. These differences were significant (P < 0.02). Also there were significant differences between the necrosis percentages of treated tumors at 1, 2 and 4 days (P < 0.02).

There was a lack of well defined necrosis zones surrounding the electrodes. The morphologic pattern of the necrotic cell mass observed is the coagulative necrosis. The dead tissue becomes both swollen and firm in consistency. Preservation of the basic profile of the coagulated cancerous cell and nuclear karyolysis were also observed. The lysed erythrocytes was also observed. This type of necrosis was accompanied by accumulation of neutrophil polymorphonuclear leucocytes.

**Figure 8:**
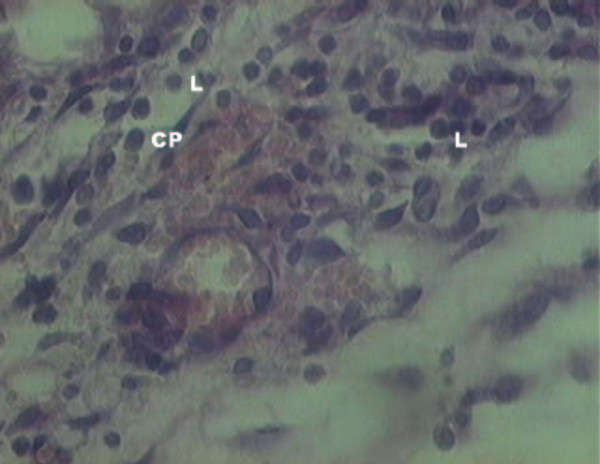
Lymphoplasmocytic infiltrate in both untreated and DEC treated tumors: plasmatic cells (CP) and lymphocytes (L). HE. × 400.

**Figure 9:**
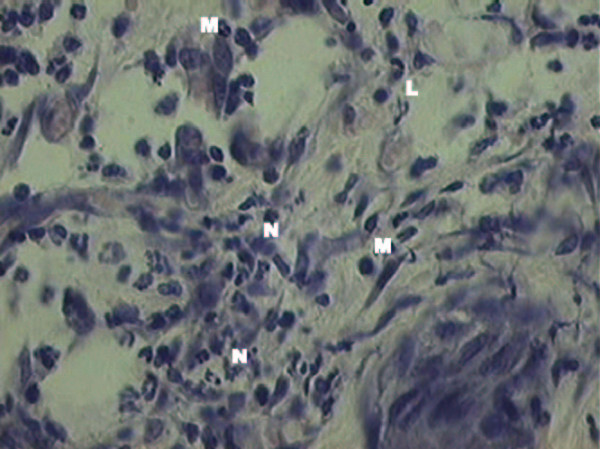
Peritumoral findings of treated tumors at 1 day after DEC treatment: leucocytes neutrophil infiltration (N), monocytes (M) and lymphocytes (L). HE. × 400.

**Figure 10:**
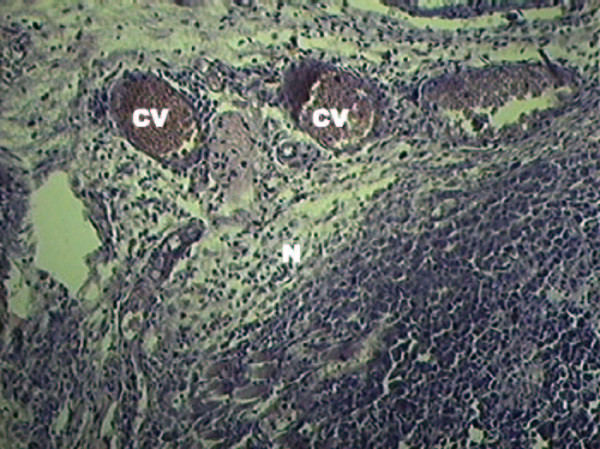
Pattern of acute inflammatory response observed during 1, 2 and 4 days after treatment: vascular congestion (CV) and neutrophils (N). HE. × 100.

Lymphocytes (L) and plasmatic cells, named CP, were observed in all the tumors in CG-A and TG-A but there were no significant differences (Table 2, Fig. 8). Neutrophil infiltration (N) and vascular congestion, named CV, were observed in all animals from the TG-A (Figs. 9 and 10). The intensity grades of these peritumoral findings were severe; however, the intensity grade of the monocyte infiltration (M) was slight to moderate in this TG-A. Edema and acute inflammatory response were observed 1, 2 and 4 days after treatment (Figs. 9 and 10). These peritumoral findings were not present in any of the animals from the CG-A (Table 2). There were significant differences (P = 0.008) between the peritumoral findings of the CG-A and TG-A.

In this experiment no mouse died from intercurrent disease during or after the treatment. Before sacrifice, the animals were active and in good physical condition with adequate body weight. They had good posture and coats of hair.

## Discussion

The results of this study demonstrated that DEC has a marked antitumor effect because a single-shot electrotherapy delivered via four platinum electrodes inserted into the base of the fibrosarcoma Sa-37 and Ehrlich murine tumors significantly retarded their growths when compared with their respective control groups. The fact that tumor regression increases with the increase of the amount of electrical charge may be explained because the induced necrosis by DEC into the tumor depends directly on its intensity, a matter that is in agreement with the results of Robertson et al. [7]. In an additional experiment was corroborated that the decrease of each treated tumor volume is due to the higher necrosis percentage induced into the tumor by DEC action. The histopathological findings made to mice 100 days post-treatment may suggest that an increase of the dose bring about an increase of the percentage of the tumor necrosis and the necrotic overlap. Also these findings confirm that the results of the pathology study were consistent with the survival study.

We believe that the necrosis is the predominant mechanism of cell death, by the cellular tumefaction (or cellular swelling), cell rupture, breakdown of organelles and acute inflammatory response observed during the first 4 days post-treatment in all treated tumors, result that agrees with that previously reported by Dodd et al. [8] and Holandino et al. [9]. Von Euler et al. [10] demonstrated that the appearance of the necrosis depends on the polarity of the electrode. The findings of necrosis observed by these researchers around anode and cathode electrodes were also observed in all treated tumors (coagulative necrosis, extravasation of blood cells, nuclear karyolysis and edema), fact that was explained because both electrodes were inserted into the tumors. On the other hand, Von Euler [11] observed both apoptosis and necrosis around the anode but only necrosis around cathode.

The necrosis may be due to the ischemia observed in all tumors treated with DEC, which could lead to an irreversible cell injury of the tumor cells and therefore to cellular death. This fact could be related with other experimental findings found after DEC treatment, such as: degradation of phospholipids, lost of high energy phosphate and increase of the intracellular calcium [7], membrane damage [5], ionic imbalance [2,12], mitochondrial alterations [9] and ischemia/reperfusion injury [13].

The prolonged acute inflammation observed during 4 days after DEC treatment may be explained by the persistent leukocyte infiltrate also observed in the peritumoral findings. This persistent leukocyte infiltrate (essential feature of the inflammatory response) becomes a harmful agent because during the chemotaxis they amplify the effects of the initial inflammatory stimulus through the liberation of potent mediators (enzymes, chemical mediators and toxic radical of oxygen) that lead to both endothelial and tissue damages. This leukocyte infiltrate may also activate the immune system [14]. In all these processes the reactive oxygen species have been shown to have an important role. In addition to these species are essential elements in the emergence of an inflammatory process [14,15]. Therefore we speculate that the oxidative burst may be the immediate cause of cell death in both tumors, although not investigated in this study.

These facts and the high necrosis percentages shown in this study may lead to the complete destruction of the solid tumor treated with DEC. The complete disappearance of the Ehrlich and fibrosarcoma Sa-37 tumors achieved for 92 and 80 C/cm^3^, respectively, may suggest that each tumor model has its threshold of electric charge from which it is completely destroyed. This threshold depends on the electric nature of the tumor and their physiological characteristics (stage, volume and histogenic characteristics). This fact explains the cure of the mice and why the tumors do not duplicate their initial volumes during the observation time (infinite DT, represented in Table 1 by ∞ symbol).

The experimental data revealed that the fibrosarcoma Sa-37 showed the higher sensitivity and curability to DEC than Ehrlich tumor and that both tumor response and survival rate of mice were DEC dependent. However, in the untreated tumors Fibrosarcoma Sa-37 showed a DT shorter than that of the Ehrlich tumor. This fact indicates the higher agressiveness of Fibrosarcoma Sa-37.

The mortality observed in all animals treated with amounts of electrical charge above 100 C/cm^3 ^could be explained by the severe damages induced by DEC in kidney and liver. Griffin et al. [12] explained this result by the induced serum electrolyte imbalance resulting from a metabolic load due to the breakdown products of the tumors.

The hemorrhage observed in the lungs of the mouse death 1 day after DEC treatment in TG1-3 may be explained by the vascular rupture and/or perforation of blood vessels due to a mechanic effect by the insertion of an electrode. The small circular necrosis also observed in this organ's mouse may be consequence of the cytotoxic action of DEC.

The uneasy and quick breathing observed in both control and treated groups, during the fixation of the mice did not have any influence in the results obtained in this study.

## Conclusions

The data presented indicate that electrotherapy with low-level DEC is feasible and effective in the treatment of the Ehrlich and fibrosarcoma Sa-37 tumors. Our results demonstrate that the sensitivity of these tumors to direct electric current and survival rates of the mice depended on both the amount of electrical charge and the type of tumor. Also the complete regression of each type of tumor is obtained for a threshold amount of electrical charge.

## Competing interests

The author(s) declare that they have no competing interests

## Authors' contributions

HCC conceived the study, and participated in its design and coordination. Also, he carried out the inoculation of the tumor cells in the mice, the measure of the tumor volumes and the survival rate of mice as well as elaborated the manuscript. MCSQ participated in the design of the study and participated in the measure of the histological findings of the organs and tumor and peritumoral findings and as well as elaborated the manuscript. LEBC carried out the inoculation of the tumor cells in the mice, conceived and participated in the design of the study and performed the statistical analysis as well as elaborated the manuscript. RNPB and DSL participated in the design of the study and contributed to elaboration of this manuscript. MFS participated in the design of the study and carried out the serology. All authors read and approved the final manuscript. OGP and TRG participated in the design of the study and contributed to elaboration of this manuscript. All authors read and approved the final manuscript. JLMF participated in the design of the study and performed the statistical analysis.

## Pre-publication history

The pre-publication history for this paper can be accessed here:



## Figures and Tables

**Table 1:** Mean doubling time

	Ehrlich Tumor				Fibrosarcoma Sa-37 tumor			
	CG1	TG1-1	TG1-2	TG1-3	CG2	TG2-1	TG2-2	TG2-3
DT^1^	2.4 ± 0.3	6.8 ± 0.7	16.9 ± 2.4	∞^3^	1.6 ± 0.2	11.2 ± 1.3	23.6 ± 3.8	∞^3^
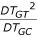	-	2.9	7.1	∞^3^	-	7.0	14.9	∞^3^

^1 ^DT (in days) is the double time of the tumors. Data are means ± standard deviation of tumors.

^2 ^DT_TG/CG _is a variable that characterizes the increase of DT in each treated group. (DT_TG_) in respect to its control group (DT_CG_) for both types of tumors.

^3 ^The symbol ∞ means infinite tumor doubling time (see Discussion).

**Table 2:** Peritumoral pathological findings The number of the mice in each experimental group is specified by n. CGA is the control group and TG1-A, TG2-A and TG3-A are the experimental subgroups of the group treated with 77 C/cm^3^at 1, 2 and 4 days after DEC treatment, respectively. Mc Nemar Test shows statistically significant differences in peritumoral findings at 1, 2 and 4 days.

Alterations found	Experimental Groups	Number of mice (% of the total) in different degrees of alteration^b^			
		
		-	+	++	+++
Lymphoplasmocytic infiltrate: Lymphocytes (L) and plasmatic cells (CP)	CGA (n = 6)	0 (0.0)	0 (0.0)	6 (100.0)	0 (0.0)
	TGA-1 day (n = 6)	0 (0.0)	0 (0.0)	6 (100.0)	0 (0.0)
	TGA-2 days (n = 6)	0 (0.0)	0 (0.0)	6 (100.0)	0 (0.0)
	TGA-4 days (n = 6)	0 (0.0)	0 (0.0)	6 (100.0)	0 (0.0)
Neutrophilic infiltrate (N)	CGA (n = 6)	6 (100.0)	0 (0.0)	0 (0.0)	0 (0.0)
	TGA-1 day(n = 6)	0 (0.0)	0 (0.0)	0 (0.0)	6 (100.0)^a^
	TGA-2 days (n = 6)	0 (0.0)	0 (0.0)	0 (0.0)	6 (100.0)^a^
	TGA-4 days (n = 6)	0 (0.0)	0 (0.0)	0 (0.0)	6 (100.0)^a^
Monocytic infiltrate (M)	CGA (n = 6)	6 (100.0)	0 (0.0)	0 (0.0)	0 (0.0)
	TGA-1 day (n = 6)	0 (0.0)	6 (100.0)^a^	0 (0.0)	0 (0.0)
	TGA-2 days (n = 6)	0 (0.0)	0 (0.0)	6 (100.0)^a^	0 (0.0)
	TGA-4 days (n = 6)	0 (0.0)	0 (0.0)	6 (100.0)^a^	0 (0.0)
Vascular congestion (CV)	CGA (n = 6)	6 (100.0)	0 (0.0)	0 (0.0)	0 (0.0)
	TGA-1 day (n = 6)	0 (0.0)	0 (0.0)	0 (0.0)	6 (100.0)^a^
	TGA-2 days (n = 6)	0 (0.0)	0 (0.0)	0 (0.0)	6 (100.0)^a^
	TGA-4 days (n = 6)	0 (0.0)	0 (0.0)	0 (0.0)	6 (100.0)^a^

^a ^P = 0.008.

^b ^Note: The signs " -, +, ++ and +++ ", represent: none, slight, moderate and severe intensity grades of alterations found, respectively.
